# International validation of a urinary biomarker panel for identification of active lupus nephritis in children

**DOI:** 10.1007/s00467-016-3485-3

**Published:** 2016-09-03

**Authors:** Eve Mary Dorothy Smith, Andrea Lyn Jorgensen, Angela Midgley, Louise Oni, Beatrice Goilav, Chaim Putterman, Dawn Wahezi, Tamar Rubinstein, Diana Ekdawy, Rachel Corkhill, Caroline Ann Jones, Stephen David Marks, Paul Newland, Clarissa Pilkington, Kjell Tullus, Michael William Beresford

**Affiliations:** 1 Department of Women’s and Children’s Health, Institute of Translational Medicine, Institute of Child Health in the Park, Alder Hey Children’s Hospital and University of Liverpool, Eaton Road, Liverpool, L12 2AP UK; 2 Department of Biostatistics, Institute of Translational Medicine, University of Liverpool, Liverpool, UK; 3 Division of Nephrology, Children’s Hospital at Montefiore and Albert Einstein College of Medicine, Bronx, NY USA; 4 Division of Rheumatology, Albert Einstein College of Medicine and Montefiore Medical Center, Bronx, NY USA; 5 Division of Pediatric Rheumatology, Children’s Hospital at Montefiore and Albert Einstein College of Medicine, Bronx, NY USA; 6 Department of Paediatric Nephrology, Alder Hey Children’s NHS Foundation Trust, Liverpool, UK; 7 Department of Paediatric Nephrology, Great Ormond Street Hospital, London, UK; 8 Biochemistry Department, Alder Hey Children’s NHS Foundation Trust, Liverpool, UK; 9 Department of Paediatric Rheumatology, Great Ormond Street Hospital, London, UK; 10 Department of Paediatric Rheumatology, Alder Hey Children’s NHS Foundation Trust, Liverpool, UK

**Keywords:** Lupus nephritis, Urine biomarkers, Glomerulonephritis, BILAG, Systemic lupus erythematosus

## Abstract

**Background:**

Conventional markers of juvenile-onset systemic lupus erythematosus (JSLE) disease activity fail to adequately identify lupus nephritis (LN). While individual novel urine biomarkers are good at detecting LN flares, biomarker panels may improve diagnostic accuracy. The aim of this study was to assess the performance of a biomarker panel to identify active LN in two international JSLE cohorts.

**Methods:**

Novel urinary biomarkers, namely vascular cell adhesion molecule-1 (VCAM-1), monocyte chemoattractant protein 1 (MCP-1), lipocalin-like prostaglandin D synthase (LPGDS), transferrin (TF), ceruloplasmin, alpha-1-acid glycoprotein (AGP) and neutrophil gelatinase-associated lipocalin (NGAL), were quantified in a cross-sectional study that included participants of the UK JSLE Cohort Study (Cohort 1) and validated within the Einstein Lupus Cohort (Cohort 2). Binary logistic regression modelling and receiver operating characteristic curve analysis [area under the curve (AUC)] were used to identify and assess combinations of biomarkers for diagnostic accuracy.

**Results:**

A total of 91 JSLE patients were recruited across both cohorts, of whom 31 (34 %) had active LN and 60 (66 %) had no LN. Urinary AGP, ceruloplasmin, VCAM-1, MCP-1 and LPGDS levels were significantly higher in those patients with active LN than in non-LN patients [all corrected *p* values (*p*_c_) < 0.05] across both cohorts. Urinary TF also differed between patient groups in Cohort 2 (*p*_c_ = 0.001). Within Cohort 1, the optimal biomarker panel included AGP, ceruloplasmin, LPGDS and TF (AUC 0.920 for active LN identification). These results were validated in Cohort 2, with the same markers resulting in the optimal urine biomarker panel (AUC 0.991).

**Conclusion:**

In two international JSLE cohorts, urinary AGP, ceruloplasmin, LPGDS and TF demonstrate an ‘excellent’ ability for accurately identifying active LN in children.

**Electronic supplementary material:**

The online version of this article (doi:10.1007/s00467-016-3485-3) contains supplementary material, which is available to authorized users.

## Introduction

Juvenile-onset systemic lupus erythematosus (JSLE) is a life-threatening multi-system autoimmune disease that displays a more aggressive course than adult onset SLE [1–3]. More renal manifestations occur in childhood, with up to 80 % of JSLE patients developing lupus nephritis (LN) within the first 5 years from diagnosis [1, 4–9]. LN is characterised by a relapsing and remitting course, requiring close surveillance and prompt treatment to prevent renal damage. Worldwide, the 5-year renal survival rate in children with LN has been shown to vary between 44 and 94 % [10–13].

Renal histology is the gold standard for diagnosing and predicating renal prognosis in LN, but only provides a snapshot of a discrete area of the kidney and is rarely repeated for monitoring purposes due to its invasive nature [14, 15]. Composite disease activity scores, such as the British Isles Lupus Assessment Group (BILAG) score or the Systemic Lupus Erythematosus Disease Activity Index (SELENA SLEDAI), and a number of traditional clinical biomarkers can be used to assess JSLE disease activity; however their role in monitoring LN within the clinic is limited [16–19].

Over recent years, numerous individual novel urinary biomarkers have been investigated for monitoring LN disease activity. These have outperformed both traditional and novel serum biomarkers, including monocyte chemoattractant protein-1 (MCP-1), neutrophil gelatinase associated lipocalin 1 (NGAL), vascular cell adhesion molecule-1 (VCAM-1) and tumour necrosis-like weak inducer of apoptosis (TWEAK) [20–26]. Using a proteomic approach, urinary transferrin (TF), ceruloplasmin, lipocalin-type prostaglandin D synthase (LPGDS), alpha-1-acid glycoprotein (AGP), albumin and albumin fragments have been shown to differentiate between children with active LN and no LN [27]. When assessed longitudinally, LPGDS, AGP and TF levels were all elevated up to 3 months before the LN flare [27].

No individual urine biomarker has achieved an ‘excellent’ predictive value [area under the receiver operating characteristic (ROC) curve (AUC) > 0.9] to date. Combining urinary biomarkers in a ‘biomarker panel’ has been shown to improve the ability to predict renal function loss in a combined paediatric/adult SLE cohort with LN [28] and relate to LN histological features [29] and activity [30].

This study therefore aimed to build on previous work [22, 25–27, 31–33] by exploring the most promising candidate urinary biomarkers to date used in combination, namely VCAM-1, MCP-1, NGAL, ceruloplasmin, TF, LPGDS and AGP in a paediatric cohort from the UK (UK JSLE Cohort Study), to assess which novel biomarker combinations can improve the identification of active LN. Since the JSLE phenotype and disease severity varies by ethnicity and race [2, 4, 34], we sought to confirm our results in a validation cohort from the USA [Einstein Lupus Cohort (ELC)] [35] in order to identify a urinary biomarker panel which is internationally applicable. Such a transatlantic comparison of a biomarker panel provides considerable strength to this study and the validation of this panel.

## Methods

### Patients

This study was based on two cross-sectional JSLE cohorts: the exploratory UK JSLE Cohort [1], which included all recruited patients from Alder Hey Children’s NHS Foundation Trust, Liverpool, and Great Ormond Street NHS Hospital for Children, London, UK. The validation cohort included ELC patients who were followed regularly at lupus clinics at the Children’s Hospital at Montefiore, Bronx, NY, USA [35]. In both cohorts, urine samples were collected during routine clinical care together with detailed demographic data, self-reported ethnicity/race data, clinical laboratory results and medication information. Disease activity data were determined using the BILAG2004 disease activity score [36, 37]. Eligible patients were diagnosed with JSLE prior to 16 years of age and met four or more of the revised American College of Rheumatology (ACR) SLE classification criteria [38]. Patients were excluded if they had a urinary tract infection or if no urine samples had been collected.

### Renal disease activity classification

Patients were categorised according to the renal domain of the BILAG2004 disease activity score, defined as follows: BILAG2004 grade A/B: severe, moderate disease respectively; grade D, inactive disease but previous system involvement; grade E, system has never been involved [37]. The composite renal BILAG score consists of six items, including proteinuria [defined in terms of urine dipstick or urine protein/albumin-to-creatinine ratio (UACR) or 24-h protein levels], deteriorating renal function [based on plasma creatinine (Cr) and glomerular filtration rate (GFR)], presence of active urinary sediment, hypertension, nephrotic syndrome and histological evidence of active nephritis in the previous 3 months, with different test score cut-offs relating to the different disease activity categories. In both cohorts, all patients with active LN had biopsy-proven LN during their disease course. Renal disease activity was therefore defined as having a renal BILAG2004 score of A or B with previous histological confirmation of LN. Non-LN was defined by a renal BILAG2004 score of D or E. This study sought to identify biomarkers that differentiate between the binary outcome of active versus no LN, therefore renal BILAG2004 C patients (where a patient had mild or improving renal disease) were excluded.

### Urine sample selection

In Cohort 1, when more than one patient’s urine sample had been collected, urine biomarkers were quantified in a single sample for inclusion within this study (cross-sectional approach). A sample from a patient with active LN (active-LN sample) was chosen for inclusion where available in order to allow as many patients with active LN as possible to contribute to the study. If a patient contributed a sample that was inactive for LN (inactive-LN sample), then the first sample collected with adequate aliquots for quantification of the whole biomarker panel was included. In Cohort 2, 23/30 study patients had an active-LN sample available and 14/30 had and inactive-LN (non-LN) sample available. Urine biomarker levels were quantified in all samples, however, 16 of these active-LN and all 14 non-LN samples contributed to the cross-sectional analysis in order to provide similar patient numbers per group. The other seven active-LN samples were subsequently included in analyses comparing urine biomarker concentrations in biopsy versus renal BILAG-defined active LN.

### Extra-renal disease activity classification

To allow assessment of biomarker levels according to whether extra-renal JSLE disease activity was present or not, patients were subdivided further as having ‘any active extra-renal involvement’ if they had a BILAG2004 of A or B in any of the remaining domains (constitutional, mucocutaneous, neuropsychiatric, musculoskeletal, cardiorespiratory, gastrointestinal, ophthalmic or haematological) or ‘no extra-renal involvement’ if they had a BILAG2004 score of D or E in all extra-renal domains. Biomarker levels were therefore compared in active/non-LN patients with and without extra-renal involvement.

### Laboratory techniques

Urine dipstick and/or microscopy and culture excluded infection. Samples were centrifuged at 2000 rpm for 10 min. Aliquots of the urine supernatant were made and stored at −80 °C until analysis. Pre-coated enzyme-linked immunosorbent assay (ELISA) kits were used to quantify urinary ceruloplasmin (Assaypro, St Charles, MO), TF (GenWay, San Diego, CA), LPGDS (BioVendor, Brno, Czech Republic), AGP and MCP-1 (R&D Systems Ltd., Minneapolis, MN). An R&D systems duo-kit (R&D Systems Ltd.) was used to quantify urinary VCAM-1 following internal validation (95 % spike recovery, 104 % linearity of dilution, co-efficients of inter/intra-assay variability 5.1 and 7.5 %, respectively). The ceruloplasmin, LPGDS, MCP-1 and AGP assays are commercially validated for use in urine and were used in accordance with the respective manufacturer’s instructions. Urinary NGAL and Cr concentrations were measured using Abbott Architect assays (Abbott Laboratories, Dallas, TX). All biomarker results were standardised for urinary Cr concentration and presented in units per milligram Cr (mgCr).

### Statistical analysis

Summary statistics for demographics (age at diagnosis, current age, gender, ethnicity), baseline clinical data (medication use and laboratory parameters) and biomarker data (ceruloplasmin, TF, LPGDS, MCP-1, VCAM-1, AGP and NGAL) were provided in terms of median values and interquartile ranges (IQR). Univariate logistic regression (quantitative data) and Pearson’s Chi-square test (binary data) were used to assess for differences in demographic and clinical factors between different patient groups. Due to the number of factors explored, a Bonferroni adjustment was applied to account for multiple testing (16 comparisons per cohort).

Mann–Whitney *U* tests with Bonferroni adjustments were used to compare biomarker concentrations between active-LN and non-LN patients (7 comparisons). Correlation between the individual urine biomarkers was assessed using Spearman’s rank correlation tests. The grading of correlation co-efficients (*r*) can vary, but for the purposes of this study 0.2–0.3 = weak/little correlation, 0.3–0.7 = moderate correlation and 0.7–1.0 = strong correlation [39]. A binary logistic regression model was fitted to assess for association between a combination of biomarkers and LN status (outcome: active-LN active = 1; non-LN JSLE = 0). All novel biomarkers (log-transformed) were included in an initial model and the ‘stepAIC’ function in R [40] applied to select a final model. This function compares models based on all possible combinations of biomarkers and chooses the model with the minimum Akaike information criterion (AIC) value. The AIC is a measure of the relative quality of a model relative to each of the other models, with a lower value meaning better quality. The AUC for the final model was calculated. Each of the remaining novel biomarkers was then added back into the final model in turn (step-wise), in order of statistical significance according to the original model including all novel biomarkers, and the AUC for each updated model calculated. This procedure allowed exploration of the effect of each biomarker on the model’s AUC, as well as an assessment of which combination of biomarkers led to the optimal AUC. This final process was repeated in the ELC validation cohort in order to determine whether the findings could be replicated. The data were then pooled to identify the optimal combined model. AUC values of 1.0–0.9, 0.9–0.8, 0.8–0.7, 0.7–0.6 and 0.6–0.5 were considered to be excellent, good, fair, poor and fail, respectively [41].

To assess the renal specificity of the urine biomarkers and whether biomarker levels vary according to whether extra-renal JSLE disease activity is present, biomarker levels in patients with ‘any active extra-renal involvement’ were compared to those with ‘no extra-renal involvement’ (Mann–Whitney *U* tests with a Bonferroni adjustment for the 7 biomarkers examined). Similarly, when comparing urinary biomarker levels in patients where a diagnosis of LN was made on the basis of recent renal biopsy results versus BILAG-defined nephritis alone, Bonferroni-adjusted Mann–Whitney *U* tests were also used. The ability of traditional biomarkers to identify active LN was investigated using binary logistic regression models for each/a combination of biomarkers (log-transformed) and LN status, and the AUC calculated.

Data analysis was undertaken using the Statistics Package for Social Sciences (SPSS; IBM Corp., Armonk, NY) version 21.0 and R version 3.1.1 [40]. Graphical illustrations were generated using GraphPad Prism version 6.0 (Graphpad Software, San Diego, CA). Where Bonferroni adjustment was made to account for multiple testing, the Bonferroni corrected *p* value, *p*_c_, is reported.

## Results

### Cohort 1—exploratory cohort (UK JSLE Cohort Study)

#### Clinical and demographic data

The UK JSLE Study cohort consisted of 61 patients with JSLE, of whom 15 (25 %) were classed as JSLE with active LN (2/15 renal BILAG score = A, 13/15 = B) and 46 (75 %) as JSLE with inactive LN (non-LN; 27/46 renal BILAG score = D, 19/46 = E). Active and non-LN JSLE patients had a median age of 15.8 [IQR 14.8–17.1] and 15.4 [IQR 13.8–17.5] years, respectively, with disease duration of 2.8 [IQR 0.7–3.9] and 2.4 [IQR 0.8–4.8] years at the time of biomarker analysis. Females comprised 86.7 % of the active-LN patients and 62.5 % of the non-LN patients. There was no difference in ethnicity between patient groups. All JSLE patients had a median of five ACR classification criteria at diagnosis [IQR 4–7]. All active-LN patients had biopsy-proven LN during their disease course, with the majority having International Society of Nephrology/Renal Pathology Society 2003 (ISN/RPS) class III (59 %) or IV (27 %) LN. Class II (7 %) and mixed class II/V (7 %) LN was seen in the remaining patients (see Table 1).

**Table 1:** Clinical, demographic and laboratory measurements at the time of urinary biomarker quantification

Variables	Exploratory Cohort 1 (UK JSLE Cohort)			Validation Cohort 2 [Einstein Lupus Cohort (USA)]		
	Active-LN^a^ (*n* = 15)	Non-LN^a^ ( *n* = 46)	*p*_c_^b^	Active-LN^a^ (*n* = 16)	Non-LN^a^ (*n* = 14)	*p*_c_^b^
Age at time of analysis (years)	16 [15–17]	15 [14–18]	ns	15 [14–17]	18 [15–19]	ns
Disease duration (years)	2.8 [0.7–3.9]	2.4 [0.8–4.8]	ns	3.1 [1.2–4.8]	1.7 [0.5–5.6]	ns
Female^c^	13 (86.7)	35 (62.5)	ns	16 (100)	10 (71)	ns
ACR^d^	5 [4–7]	5 [4–7]	ns	5 [5.0–5.8]	5 [4.5–6.0]	ns
Ethnicity^e^						
Caucasian	2 (13)	23 (50)		0 (0)	0 (0)	
African^f^	3 (20)	5 (11)		11 (69)	5 (36)	
Hispanic	0 (0)	0 (0)		5 (31)	8 (57)	
Caribbean	2 (13)	2 (4)	ns	0 (0)	0 (0)	ns
Mixed race	3 (20)	0 (0)		0 (0)	0 (0)	
Indian	3 (21)	11 (24)		0 (0)	1 (7)	
Chinese	2 (13)	5 (11)		0 (0)	0 (0)	
Medication use^g^						
Prednisolone	12 (80)	21 (46)	ns	14 (88)	12 (86)	ns
Mycophenolate mofetil	11 (73)	19 (41)	ns	7 (44)	3 (21)	ns
Cyclophosphamide ever	3 (20)	2 (4)	ns	9 (56)	4 (29)	ns
Rituximab ever	5 (33)	0 (0)	0.02	6 (38)	5 (36)	ns
ACEi/AT2	4 (27)	6 (13)	ns	10 (63)	1 (7)	0.03
Glomerular filtration rate^h^	100 [70–112]	116 [105–127]	ns	126 [90–160]	110 [100–123]	ns
Urinary albumin-to-creatinine (Cr) ratio (mg/mmolCr)	92 [23–153]	1 [1–2]	<0.01	555 [137–2059]	9 [3–19]	0.03
Serum creatinine (μmol/L)	57 [50–86]	53 [46–61]	ns	53 [44–71]	66 [62–73]	ns
dsDNA (IU/L)	48 [15–263]	2 [0.1–52]	ns	156 [96–179]	87 [23–178]	ns
C3 (g/L)	1.0 [0.5–1.2]	1.1 [1–1.2]	ns	0.8 [0.7–1.0]	1.0 [0.8–1.2]	ns
ESR (mm/h)^i^	55 [20–90]	9 [3–23]	<0.01	−	−	−

Data are expressed as median values with the interquartile range (IQ) in square brackets, or as numbers with the percentage in parenthesis, as appropriate

JSLE, Juvenile-onset systemic lupus erythematosus; LN, lupus nephritis; ACEi/AT2, angiotensin-converting enzyme inhibitor/angiotensin 2 blocker; dsDNA, anti-double-stranded DNA antibody; C3, complement component 3; ESR, erythrocyte sedimentation rate

^a^Classification of the patients into active-LN/Non-LN groups is described in section Urine sample selection

^b^ *p* values are Bonferroni-corrected *p* values (*p*_c_) from Chi-squared tests or univariate binary regression, as appropriate. ns = *p*_c_ > 0.05

^c^Gender data missing on one Cohort 1 patient

^d^ACR, Number of American College of Rheumatology criteria for systemic lupus erythematosus (SLE) fulfilled at diagnosis

^e^Self-reported ethnicity data shown

^f^Within Cohort 2, African American patients were also included in this category

^g^Current medication use is described for regular medications; those medications taken in courses/intermittently are described as having been used ‘ever’

^h^mls/min

^i^ESR was not routinely measured in Cohort 2

Compared to non-LN patients, more active-LN patients had received rituximab (*p*_c_ < 0.05), but the use of other medications did not differ significantly between the patient groups. Of the laboratory parameters investigated, the UACR and erythrocyte sedimentation rate (ESR) were significantly higher in the active-LN patients (all *p*_c_ < 0.05) (see Table 1).

### Novel urinary biomarkers

**Fig. 1:**
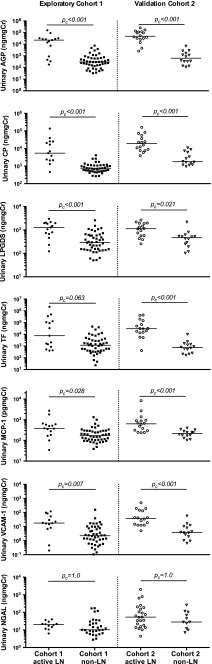
Distribution of biomarker concentrations in active-/non-lupus nephritis (*LN*) patients with juvenile-onset systemic lupus erythematosus (JSLE) from Cohorts 1 (UK JSLE Cohort) and 2 [Einstein Lupus Cohort (ELC)]. *Horizontal line* Median value for each group. Mann–Whitney *U* tests were used to compare the distribution of biomarker concentrations between patient groups within each cohort. A Bonferroni adjustment was applied to account for multiple testing. Corrected *p* values (*p*_*c*_) are reported. Vascular cell adhesion molecule-1 (*VCAM-1*) biomarker data were not available from one active-LN patient from Cohort 1; neutrophil gelatinase-associated lipocalin (*NGAL*) data were not available from three active-LN and 15 non-LN patients from Cohort 1. *AGP* Alpha-1-acid glycoprotein, *CP* ceruloplasmin, *LPGDS* lipocalin-like prostaglandin D synthase, *TF* transferrin, *MCP-1* monocyte chemoattractant protein 1, *Cr* creatinine. See section Urine sample selection for definition of active-/non-LN

Figure 1 depicts the distribution of novel urinary biomarker concentrations standardised to urinary Cr in patients with active LN and no LN. Patients with active LN had significantly higher urinary concentrations of AGP, ceruloplasmin, VCAM-1, MCP-1 and LPGDS than non-LN patients [all *p*_c_ < 0.05; see Fig. 1 and Electronic Supplementary Material (ESM) 1]. Urinary TF and NGAL concentrations did not differ significantly between the patient groups (*p*_c_ = 0.06 and 1.0, respectively; see Fig. 1 and ESM 1). LPGDS and AGP were strongly correlated (*r* = 0.71). All other biomarker combinations were moderately correlated (*r* = 0.3–0.7) except for LPGDS + TF and MCP-1 + TF, which were weakly correlated (*r* < 0.3; see ESM 2 for further details).

**Fig. 2:**
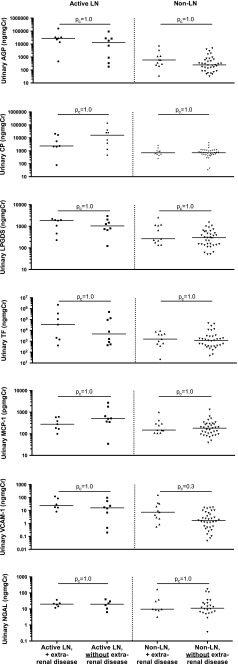
Urine biomarker concentrations in active-/non- lupus nephritis (LN) patients with/without extra-renal juvenile-onset systemic lupus erythematosus (JSLE) activity. Biomarker concentrations were standardised to urinary creatinine and expressed as median values. *Horizontal line* Median value for each group. Mann–Whitney *U* tests were used to compare biomarker concentrations between patient groups. A Bonferroni adjustment was applied to account for multiple testing. Corrected *p* values (*p*_*c*_) are reported. Vascular cell adhesion molecule-1 (VCAM-1) measurement is missing from one patient; neutrophil gelatinase-associated lipocalin (NGAL) data were not available from three active-LN and 15 non-LN patients

Urine biomarker levels did not differ between non-LN patients who had previous LN (renal-BILAG score D) and those with no previous renal involvement (renal-BILAG score E; all *p*_c_ > 0.05). Similarly, there was no difference between patients with severe or moderate active LN (renal-BILAG score A/B, respectively; all *p*_c_ > 0.05; see ESM 3). There was also no significant difference in urinary biomarker levels depending on the presence or absence of extra-renal involvement (see Fig. 2).

On fitting a binary logistic regression model including all novel biomarkers and subsequently applying the ‘stepAIC’ function in R [40], the final model included both AGP and ceruoplasmin (see Table 2). The AUC for this final model was 0.88. On addition of LPGDS, the AUC increased to 0.90, increasing further to 0.92 upon the addition of TF. The addition of VCAM-1 and MCP-1 into the model, however, did not increase the AUC (see Table 3).

**Table 2:** Binary logistic regression models initially including all biomarkers and after variable selection for Cohort 1

Biomarkers	Model including all biomarkers^a^		
	Coefficient	Standard error	*p* value
AGP	0.692	0.35	0.047
CP	0.551	0.36	0.127
VCAM-1	−0.228	0.38	0.553
LPGDS	0.870	0.76	0.254
MCP-1	−0.046	0.86	0.957
TF	0.256	0.23	0.275
	Model after variable selection^b^		
AGP	0.782	2.84	0.004
CP	0.602	0.34	0.080

AGP, Alpha-1-acid glycoprotein; CP, ceruloplasmin; VCAM-1, vascular cell adhesion molecule-1; LPGDS, lipocalin-like prostaglandin D synthase; MCP-1, monocyte chemoattractant protein 1; TF, transferrin

*AGP* alpha-1-acid glycoprotein

^a^59 Cohort 1 patients included in the exploratory novel biomarker models including VCAM-1 due to a missing measurements

^b^Model selected after applying the ‘stepAIC’ function in R

**Table 3:** Effect on the area under the receiver operating characteristic curve of adding biomarkers to the regression model in Cohort 1 and 2 separately or together

Biomarker combinations included in the binary logistic regression models	Cohort 1^a^	Cohort 2^b^	Cohorts 1 and 2 together
AGP + CP	0.881	0.982	0.935
AGP + CP + LPGDS	0.900	0.982	0.941
AGP + CP + LPGDS + TF	0.920	0.991	0.949
AGP + CP + LPGDS + TF + VCAM-1	0.920	0.987	0.952
AGP + CP + LPGDS + TF + VCAM-1 + MCP-1	0.920	NA^c^	0.949

Values on given as the area under the receiver operating characteristic (ROC) curve (AUC)

*AGP* alpha-1-acid glycoprotein, *CP* ceruloplasmin, *LPGDS* lipocalin-like prostaglandin D synthase, *TF* transferrin, *VCAM-1* vascular cell adhesion molecule-1, *MCP-1* monocyte chemoattractant protein 1

^a^59 Cohort 1 patients were included in the novel biomarker models including VCAM-1 due to missing biomarker measurements

^b^30 patients were included in Cohort 2 novel biomarker models

^c^Not available. Patient number (*n* = 30) precludes fitting of a model including all biomarkers

### Cohort 2—validation cohort (Einstein Lupus Cohort)

#### Clinical and demographic data

The validation cohort consisted of 30 JSLE patients of whom 16 (53 %) were classed as active-LN (11/16 renal BILAG score = A, 5/16 = B) and 14 (47 %) were classified as non-LN JSLE patients (6/16 renal BILAG score = D, 8/16 = E). Active- and non-LN JSLE patients had a median age of 15 and 18 years, respectively, with a respective disease duration of 3.1 and 1.7 years at the time of biomarker analysis. Females constituted 100 % of the active-LN patients and 71 % of the non-LN patients. Both JSLE patient groups had a median of five ACR classification criteria at diagnosis. ELC patients were largely African/African American (53 %) and Hispanic (43 %), whereas UK JSLE Cohort patients were predominatly Caucasian (41 %) and Indian (23 %). All active-LN patients had biopsy-proven LN during their disease course, with the ISN/RPS 2003 classes as follows; class III = 19 %, class IV = 19 %, class V = 31 %, mixed class III/V = 31 %. Both groups of patients had a median of five ACR classification criteria at diagnosis. Active-LN and non-LN patients differed significantly in terms of their UACR and use of angiotensin-converting enzyme inhibitors (ACEi)/angiotensin 2 blockers (AT2) (both *p* < 0.05, see Table 1).

### Novel urine biomarkers

Figure 1 shows the distribution of novel urinary biomarker concentrations in Cohort 2, relative to Cohort 1 patients. Patients with active LN had significantly higher urinary concentrations of AGP, ceruloplasmin, LPGDS, TF, MCP-1 and VCAM-1 than non-LN patients (all *p*_c_ < 0.05). NGAL levels did not differ between patient groups in either cohort (*p*_c_ = 1.0). Ceruloplasmin and MCP-1, AGP, TF were all strongly correlated. LPGDS was also strongly correlated with AGP and VCAM-1. AGP was strongly correlated with VCAM-1 and TF (all *r* > 0.7). All other biomarker combinations were moderately correlated (*r* = 0.3–0.7; see ESM 2 for further details).

**Fig. 3:**
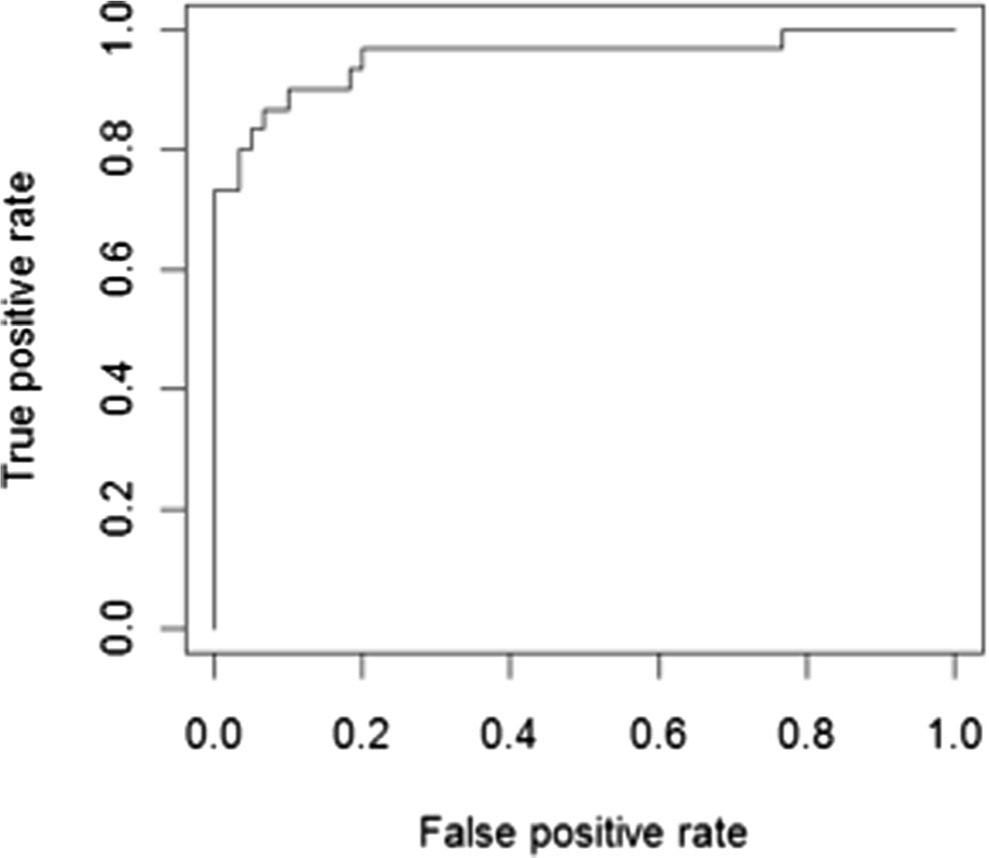
The receiver operating characteristic (ROC) cure generated from the optimal binary logistic regression model when data from both cohorts were combined. Optimal model includes Alpha-1-acid glycoprotein (AGP), ceruloplasmin, lipocalin-like prostaglandin D synthase (LPGDS), transferrin (TF) and vascular cell adhesion molecule-1 (VCAM-1) [area under the ROC curve (AUC) 0.952]

A binary logistic regression model was fitted with Cohort 2 data, adding the data on each variable in a stepwise manner one at a time in the same order as was done for Cohort 1. The model including AGP, ceruoplasmin, LPGDS and TF again produced the optimal AUC (0.991). As a combination of biomarkers led to excellent identification of active LN in both cohorts, AUCs were also calculated for both cohort datasets combined (see Table 3). A combined Cohort 1 and Cohort 2 model, including AGP, ceruoplasmin, LPGDS and TF, again gave excellent AUC (0.949); however adding VCAM-1 slightly improved the AUC further (0.952). The ROC generated by this optimal Cohort 1 and 2 model is shown in Fig. 3.

### Urine biomarker concentrations in biopsy versus renal BILAG-defined active LN

**Fig. 4:**
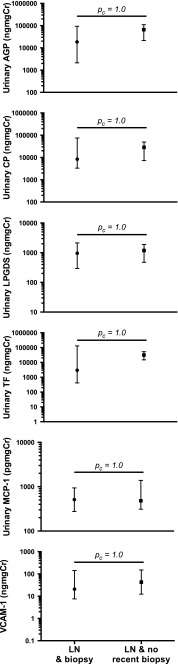
Urine biomarker concentrations in Cohort 2 patients with LN and no recent biopsy (BILAG-defined active LN; *n* = 11) versus patients with biopsy-defined active LN (*n* = 12). *Closed symbols* Median, *Whiskers* interquartile range. British Isles Lupus Assessment Group (BILAG), lupus nephritis (LN)

Urine biomarker levels from 12 samples from Cohort 2 patients which were taken at the time of or within 6 weeks of renal biopsy were compared with those of 11 patient samples with a current composite renal BILAG score-based diagnosis of active LN (but a previous history of having had biopsy-defined active LN). Urinary AGP, ceruloplasmin, LPGDS, TF, MCP-1 and VCAM-1 levels did not differ significantly between the two groups of active-LN patients (all *p*_c_ = 1.0; see Fig. 4). Urine samples from Cohort 1 patients were not available close to the time of renal biopsy; therefore, comparable groups were not available for inclusion in these analyses. The study was underpowered to assess for differences in any of the urinary biomarkers according to ISN/RPS 2003 subclass.

### Ability of traditional biomarkers to identify active LN

Traditional biomarkers which do not contribute to the composite renal BILAG score were assessed for their ability to identify active LN. ESR was the best traditional biomarker, with a fair AUC of 0.796 (ESR was only measured routinely within cohort 1). Complement component 3 (C3) and double-stranded DNA showed a poor ability to identify active LN in both cohorts (AUC from 0.617 to 0.645). C4 performed the worst, with an AUC of 0.593 and 0.482 in Cohort 1 and 2, respectively. Inclusion of all traditional biomarkers together in a regression model did not improve the AUC. Addition of ESR, the best traditional biomarker, to the optimal UK novel biomarker combination, including AGP, LPGDS, TF and ceruloplasmin, did not improve the AUC further (AUC 0.910; see TableI4).

**Table 4:** Area under the ROC curve values corresponding to the ability of traditional biomarkers to identify active lupus nephritis alone and in combination with novel biomarkers

Traditional biomarkers	AUC	
	Cohort 1	Cohort 2
dsDNA	0.617	0.643
C3	0.645	0.638
C4	0.593	0.482
ESR	0.796	NA^a^
All traditional biomarkers	0.783	0.670^b^
Optimal novel biomarker combination (AGP + CP + LPGDS + TF) + ESR	0.910	NA^a^

AUC values obtained from logistic regression model probabilities for each traditional biomarker and all biomarkers together

*dsDNA* double strand DNA, *ESR* erythrocyte sedimentation rate, *AGP* Alpha-1-acid glycoprotein, *CP* ceruloplasmin, *LPGDS* lipocalin-like prostaglandin D synthase, *TF* transferrin

^a^Not available; ESR was not routinely measured in Cohort 2

^b^ESR data missing from the Cohort 2 model

## Discussion

To optimise effective management of LN, readily available and easily measured biomarkers are urgently needed within clinical practice. Early diagnosis and prompt treatment of LN can improve long-term renal survival [18]. The invasive nature of renal biopsy limits its clinical utility, especially in childhood. By simultaneously measuring urinary AGP, ceruloplasmin, VCAM-1, TF, LPGDS, MCP-1 and NGAL at a single patient visit in two ethnically diverse cohorts of JSLE patients, the aim of this study was to derive and internationally validate a biomarker panel which could improve identification of active LN, over and above individual biomarkers. Across both cohorts we have demonstrated an optimal urine biomarker combination that includes AGP, ceruoplasmin, LPGDS and TF with excellent AUC values for active LN identification (AUC 0.920 and 0.991 for Cohorts 1 and 2, respectively). Furthermore, the presence of extra-renal disease activity does not appear to influence the accuracy of this panel of urine biomarkers. This is therefore the first LN urine biomarker panel study to include a exploratory and validation cohort, providing a firm foundation for future development of a clinical urine biomarker panel test.

Previous studies complementing our work have focussed on identifying biomarker combinations reflective of LN histological subtypes in patients with biopsy-proven LN. Brunner et al. investigated 28 childhood-onset and 48 adult-onset SLE patients, assessing biomarker combinations differentiating biopsy-defined activity, chronicity or membranous LN in samples taken within 2 months of biopsy. The best predictive ability detected by these authors was for LN activity, when MCP-1, AGP, ceruloplasmin and the urine protein-to-creatinine ratio were considered together (AUC 0.850) [29]. Within the UK JSLE Cohort and the ELC, we have demonstrated stronger AUC values (0.920 and 0.991, respectively) for the identification of active LN with the combination of urinary AGP, ceruloplasmin, LPGDS and TF. This result supports the importance of a combination approach to urinary biomarkers in LN, in these JSLE cohorts. In our present study, when the results from both the UK Cohort and ELC are pooled, VCAM-1 adds to the diagnostic ability of the above biomarker panel, indicating that further investigation of the role of VCAM-1 in combination with other biomarkers for discriminating active LN in children is required. The UK JSLE Cohort consisted of predominately Caucasian and Indian patients, whereas the ELC cohort comprised mainly African American and Hispanic patients. Notably, African and African American patients often have more severe kidney involvement in SLE [4, 34, 42]. Interestingly, within our study the optimal biomarker panel performed even better in the validation ELC than in the exploratory UK JSLE Cohort.

More recently, Brunner et al. have looked at additional biomarkers in samples taken at the time of biopsy from 47 children with ISN/RPS class II–V LN [30]. These authors demonstrated that NGAL, MCP-1, ceruloplasmin, adiponectin, hematopexin and kidney injury molecule-1 were the best predictors of LN activity status as assessed by the National Institute for Health Activity Index (NIH-AI), leading them to propose a biomarker-based Renal Activity Index for Lupus (RAIL) algorithm [30]. Our current study examined a sub-set of these markers for their ability to identify BILAG-defined active LN rather than NIH-AI status. The promising results of Brunner et al. [30] require further validation in larger prospective, multi-ethnic cohorts. In contrast to the markers validated in our current study, it remains unclear whether the biomarkers proposed by Brunner et al. [30] would be able to differentiate patients with active LN from those with inactive LN, as all patients in the their study had definite biopsy-defined LN.

Our data demonstrate the key utility of urinary biomarkers in monitoring LN. We have demonstrated and validated an excellent panel of biomarkers which differentiate JSLE patients with active LN and no current LN. As discussed above, Brunner et al. [30] have also proposed a distinct biomarker panel which accurately correlates with NIH-AI status. A large international prospective study or clinical trial is therefore warranted. This would longitudinally assess the biomarkers validated in the current study for initial identification of active LN, followed by assessment of LN severity using the additional markers included in the RAIL as a proxy for histological changes. An international collaborative study will most probably be needed to be sufficiently powered given the multiplicity of biomarkers studied, distinct kidney biopsy features seen and the ethnic differences seen in JSLE severity.

In our current study we could not demonstrate a significant difference in urinary NGAL levels between those patients with active LN and those with non-LN in either the UK Cohort Study or the ELC. This is in contrast with previous work which has shown NGAL to be highly sensitive/specific for the identification of biopsy-proven LN in children [26]. These results may be explained by differences in the timing of the sample and the outcome measures used. Urinary NGAL has previously been shown to be a useful predictor of impending flare in both the UK JSLE Cohort [26] and in an adult SLE study of the ELC which included a University College London validation cohort [43]. Kiani et al. were also unable to detect an association between urinary NGAL and LN in a prospective study that included 107 adult SLE patients [44]. These observations may be due to urinary NGAL levels peaking before flares and then receding before the event becomes clinically detectable [45]. Urinary NGAL has also been demonstrated as a marker of renal damage in LN [46], which may also explain why patients with a history of biopsy-proven LN have higher urinary NGAL levels. These observations suggest that NGAL requires further testing longitudinally as part of a urine biomarker panel despite the results seen in the current study, as it may able to predict active nephritis and inactive nephritis occurrence.

It is interesting to consider the origin and renal specificity of the novel biomarkers. AGP belongs to the immunocalin family, a group of immunomodulatory binding proteins. It is mainly produced by the liver but has also been reported in other cell types (macrophages [47], endothelial cells [48] and monocytes [49]). In active LN, increased production of AGP as part of the acute phase response, coupled with AGP production by cells infiltrating the kidney, may be responsible for the high urinary levels demonstrated. TF and ceruloplasmin are plasma proteins, primarily responsible for carrying iron and copper, respectively. Differing from albumin in terms of their molecular radii and isoelectric points, urinary ceruloplasmin and TF have been shown to predict the onset of microalbuminuria in diabetic nephropathy [50]. LPGDS, a member of the lipocalin superfamily responsible for prostaglandin D2 production, is similar to albumin in terms of chemical properties, but it is much smaller [51]. In type-2 diabetes, urinary LPGDS has been shown to increase in the early stages of kidney injury [52]. Urinary VCAM-1 levels have previously been shown to be higher than blood levels, suggesting that the inflamed kidney may represent an important source of urinary VCAM-1 [33].

Certain limitations of our study warrant recognition and should be addressed in future work. As our definition of active LN was based on the composite renal BILAG score, calculated from proteinuria, GFR, blood pressure, active urine sediment, plasma creatinine and recent biopsy findings, we could not directly compare such traditional markers with the novel urinary biomarkers studied. Due to the cross-sectional nature of this study we are unable to comment on the relationship of such biomarkers with other stages of the fluctuating LN disease course (e.g. prediction of flare/remission). Validation in a larger, longitudinal, prospectively collected study is therefore necessary, including children and young people with the full range of mild, severe and inactive disease phenotypes from a range of patient cohorts (including Asian and African cohorts). With further prospective validation, it may become apparent that fewer biomarkers together can produce acceptable accuracy for active LN identification (e.g. AGP and ceruloplasmin) due to the level of correlation seen between biomarkers (especially for Cohort 2). This would potentially make it a simpler point-of-care testing device for biomarker quantification. Concurrent investigation of the role of such biomarkers in vitro or in LN mouse models will also help to improve understand of LN pathophysiology.

## Conclusions

Patients with JSLE have significant renal involvement and the potential to develop irreversible renal damage as the result of LN relapses that are either unrecognised, not identified early enough or not treated sufficiently [4, 53]. This study has demonstrated and validated a renal-specific excellent novel urine biomarker panel for the recognition of active LN in two ethnically diverse JSLE populations, thereby providing considerable strength to these findings. Further validation in larger, longitudinal, prospectively collected studies is required to define biomarker profiles that predict LN relapses and response to treatment. It is anticipated that a future urinary biomarker point-of-care testing device will help to improve the renal outcomes for JSLE patients through biomarker-led renal monitoring in routine clinical practice.

## Electronic supplementary material

Below is the link to the electronic supplementary material.

On-line resource 1 Urine biomarker concentrations standardised to urinary creatinine in active and non-LN patients from both cohorts (DOCX 99 kb)

On-line resource 2 Correlation between urine biomarkers in cohorts 1 and 2 (DOCX 88 kb)

On-line resource 3 Urine biomarker concentrations (standardised to urinary creatinine) according to renal BILAG score in patients from the UK exploratory cohort. (DOCX 115 kb)

## References

[1] Watson L, Leone V, Pilkington C, Tullus K, Rangaraj S, McDonagh JE, Gardner-Medwin J, Wilkinson N, Riley P, Tizard J, Armon K, Sinha MD, Ioannou Y, Archer N, Bailey K, Davidson J, Baildam EM, Cleary G, McCann LJ, Beresford MW (2012). Disease activity, severity, and damage in the UK Juvenile-Onset Systemic Lupus Erythematosus Cohort. Arthritis Rheum.

[2] Tucker LB, Uribe AG, Fernandez M, Vila LM, McGwin G, Apte M, Fessler BJ, Bastian HM, Reveille JD, Alarcon GS (2008). Adolescent onset of lupus results in more aggressive disease and worse outcomes: results of a nested matched case–control study within LUMINA, a multiethnic US cohort (LUMINA LVII). Lupus.

[3] Mina R, Brunner HI (2010). Pediatric lupus-are there differences in presentation, genetics, response to therapy, and damage accrual compared with adult lupus?. Rheum Dis Clin N Am.

[4] Hiraki LT, Lu B, Alexander SR, Shaykevich T, Alarcon GS, Solomon DH, Winkelmayer WC, Costenbader KH (2011). End-stage renal disease due to lupus nephritis among children in the US, 1995–2006. Arthritis Rheum.

[5] Tucker LB, Menon S, Schaller JG, Isenberg DA (1995). Adult- and childhood-onset systemic lupus erythematosus: a comparison of onset, clinical features, serology, and outcome. Br J Rheumatol.

[6] Hiraki LT, Benseler SM, Tyrrell PN, Hebert D, Harvey E, Silverman ED (2008). Clinical and laboratory characteristics and long-term outcome of pediatric systemic lupus erythematosus: a longitudinal study. J Pediatr.

[7] Font J, Cervera R, Espinosa G, Pallares L, Ramos-Casals M, Jimenez S, Garcia-Carrasco M, Seisdedos L, Ingelmo M (1998). Systemic lupus erythematosus (SLE) in childhood: analysis of clinical and immunological findings in 34 patients and comparison with SLE characteristics in adults. Ann Rheum Dis.

[8] Barron KS, Silverman ED, Gonzales J, Reveille JD (1993). Clinical, serologic, and immunogenetic studies in childhood-onset systemic lupus erythematosus. Arthritis Rheum.

[9] Appel AE, Sablay LB, Golden RA, Barland P, Grayzel AI, Bank N (1978). The effect of normalization of serum complement and anti-DNA antibody on the course of lupus nephritis: a two year prospective study. Am J Med.

[10] Hagelberg S, Lee Y, Bargman J, Mah G, Schneider R, Laskin C, Eddy A, Gladman D, Urowitz M, Hebert D, Silverman E (2002). Longterm followup of childhood lupus nephritis. J Rheumatol.

[11] Sun L, Xu H, Liu HM, Zhou LJ, Cao Q, Shen Q, Fang XY (2011). Long-term follow-up of 101 cases with pediatric lupus nephritis in a single center in Shanghai. Zhonghua Er Ke Za Zhi.

[12] Lee BS, Cho HY, Kim EJ, Kang HG, Ha IS, Cheong HI, Kim JG, Lee HS, Choi Y (2007). Clinical outcomes of childhood lupus nephritis: a single center’s experience. Pediatr Nephrol.

[13] Ataei N, Haydarpour M, Madani A, Esfahani ST, Hajizadeh N, Moradinejad MH, Gholmohammadi T, Arbabi S, Haddadi M (2008). Outcome of lupus nephritis in Iranian children: prognostic significance of certain features. Pediatr Nephrol.

[14] Preda A, Van Dijk LC, Van Oostaijen JA, Pattynama PM (2003). Complication rate and diagnostic yield of 515 consecutive ultrasound-guided biopsies of renal allografts and native kidneys using a 14-gauge Biopty gun. Eur Radiol.

[15] Blake KD, Madden S, Taylor BW, Rees L (1996). Psychological and clinical effects of renal biopsy performed using sedation. Pediatr Nephrol.

[16] Faurschou M, Starklint H, Halberg P, Jacobsen S (2006). Prognostic factors in lupus nephritis: diagnostic and therapeutic delay increases the risk of terminal renal failure. J Rheumatol.

[17] Esdaile JM, Levinton C, Federgreen W, Hayslett JP, Kashgarian M (1989). The clinical and renal biopsy predictors of long-term outcome in lupus nephritis: a study of 87 patients and review of the literature. Q J Med.

[18] Esdaile JM, Joseph L, MacKenzie T, Kashgarian M, Hayslett JP (1994). The benefit of early treatment with immunosuppressive agents in lupus nephritis. J Rheumatol.

[19] Illei GG, Tackey E, Lapteva L, Lipsky PE (2004). Biomarkers in systemic lupus erythematosus: II. Markers of disease activity. Arthritis Rheum.

[20] Schwartz N, Rubinstein T, Burkly LC, Collins CE, Blanco I, Su L, Hojaili B, Mackay M, Aranow C, Stohl W, Rovin BH, Michaelson JS, Putterman C (2009). Urinary TWEAK as a biomarker of lupus nephritis: a multicenter cohort study. Arthritis Res Ther.

[21] Abujam B, Cheekatla S, Aggarwal A (2013). Urinary CXCL-10/IP-10 and MCP-1 as markers to assess activity of lupus nephritis. Lupus.

[22] Howe HS, Kong KO, Thong BY, Law WG, Chia FL, Lian TY, Lau TC, Chng HH, Leung BP (2012). Urine sVCAM-1 and sICAM-1 levels are elevated in lupus nephritis. Int J Rheum Dis.

[23] Suzuki M, Wiers KM, Klein-Gitelman MS, Haines KA, Olson J, Onel KB, O’Neil K, Passo MH, Singer NG, Tucker L, Ying J, Devarajan P, Brunner HI (2008). Neutrophil gelatinase-associated lipocalin as a biomarker of disease activity in pediatric lupus nephritis. Pediatr Nephrol.

[24] Abd-Elkareem MI, Al Tamimy HM, Khamis OA, Abdellatif SS, Hussein MR (2010). Increased urinary levels of the leukocyte adhesion molecules ICAM-1 and VCAM-1 in human lupus nephritis with advanced renal histological changes: preliminary findings. Clin Exp Nephrol.

[25] Singh S, Wu T, Xie C, Vanarsa K, Han J, Mahajan T, Oei HB, Ahn C, Zhou XJ, Putterman C, Saxena R, Mohan C (2012). Urine VCAM-1 as a marker of renal pathology activity index in lupus nephritis. Arthritis Res Ther.

[26] Watson L, Tullus K, Pilkington C, Chesters C, Marks SD, Newland P, Jones CA, Beresford MW (2013). Urine biomarkers for monitoring juvenile lupus nephritis: a prospective longitudinal study. Pediatr Nephrol.

[27] Suzuki M, Wiers K, Brooks EB, Greis KD, Haines K, Klein-Gitelman MS, Olson J, Onel K, O’Neil KM, Silverman ED, Tucker L, Ying J, Devarajan P, Brunner HI (2009). Initial validation of a novel protein biomarker panel for active pediatric lupus nephritis. Pediatr Res.

[28] Abulaban K, Brunner H, Nelson SL, Bennett M, Ying J, Song H, Kimmel P, Kusek J, Feldman H, Ramachandran V, Rovin BH (2014). Urine biomarkers role in predicting the future development of renal functional loss with lupus nephritis in children and adults. Arthritis Rheum.

[29] Brunner HI, Bennett MR, Mina R, Suzuki M, Petri M, Kiani AN, Pendl J, Witte D, Ying J, Rovin BH, Devarajan P (2012). Association of noninvasively measured renal protein biomarkers with histologic features of lupus nephritis. Arthritis Rheum.

[30] Brunner HI, Bennett M, Abulaban K, Klein-Gitelman M, O’Neil K, Tucker L, Ardoin S, Rouster-Stevens K, Onel K, Singer N, Eberhard BA, Jung L, Imundo L, Wright T, Witte D, Rovin B, Ying J, Devarajan P (2015) Development of a novel renal activity index of lupus nephritis in children & young adults. Arthritis Care Res (Hoboken) 68(7):1003–1011 10.1002/acr.22762 PMC4834060 26473509

[31] Molad Y, Miroshnik E, Sulkes J, Pitlik S, Weinberger A, Monselise Y (2002). Urinary soluble VCAM-1 in systemic lupus erythematosus: a clinical marker for monitoring disease activity and damage. Clin Exp Rheumatol.

[32] Watson L, Midgley A, Pilkington C, Tullus K, Marks S, Holt R, Jones C, Beresford M (2012). Urinary monocyte chemoattractant protein 1 and alpha 1 acid glycoprotein as biomarkers of renal disease activity in juvenile-onset systemic lupus erythematosus. Lupus.

[33] Wu T, Xie C, Wang HW, Zhou XJ, Schwartz N, Calixto S, Mackay M, Aranow C, Putterman C, Mohan C (2007). Elevated urinary VCAM-1, P-selectin, soluble TNF receptor-1, and CXC chemokine ligand 16 in multiple murine lupus strains and human lupus nephritis. J Immunol.

[34] Nee R, Martinez-Osorio J, Yuan CM, Little DJ, Watson MA, Agodoa L, Abbott KC (2015). Survival Disparity of African American Versus Non-African American Patients With ESRD Due to SLE. Am J Kidney Dis.

[35] Schwartz N, Su L, Burkly LC, Mackay M, Aranow C, Kollaros M, Michaelson JS, Rovin B, Putterman C (2006). Urinary TWEAK and the activity of lupus nephritis. J Autoimmun.

[36] Marks SD, Pilkington C, Woo P, Dillon MJ (2004). The use of the British Isles Lupus Assessment Group (BILAG) index as a valid tool in assessing disease activity in childhood-onset systemic lupus erythematosus. Rheumatology.

[37] Isenberg DA, Rahman A, Allen E, Farewell V, Akil M, Bruce IN, D’Cruz D, Griffiths B, Khamashta M, Maddison P, McHugh N, Snaith M, Teh LS, Yee CS, Zoma A, Gordon C (2005). BILAG 2004. Development and initial validation of an updated version of the British Isles Lupus Assessment Group’s disease activity index for patients with systemic lupus erythematosus. Rheumatology.

[38] Tan EM, Cohen AS, Fries JF, Masi AT, McShane DJ, Rothfield NF, Schaller JG, Talal N, Winchester RJ (1982). The 1982 revised criteria for the classification of systemic lupus erythematosus. Arthritis Rheum.

[39] Harris M, Taylor G (2014). Medical statistics made easy 3.

[40] R Core Team (2013) R: a language and environment for statistical computing. R Foundation for Statistical Computing, Vienna. Available at:http://www.r-project.org/. Accessed 31 Aug 2015

[41] Akobeng AK (2007). Understanding diagnostic tests 3: Receiver operating characteristic curves. Acta Paediatr.

[42] Dall’Era M, Levesque V, Solomons N, Truman M, Wofsy D (2015). Identification of clinical and serological factors during induction treatment of lupus nephritis that are associated with renal outcome. Lupus Sci Med.

[43] Rubinstein T, Pitashny M, Levine B, Schwartz N, Schwartzman J, Weinstein E, Pego-Reigosa JM, Lu TY, Isenberg D, Rahman A, Putterman C (2010). Urinary neutrophil gelatinase-associated lipocalin as a novel biomarker for disease activity in lupus nephritis. Rheumatology.

[44] Kiani AN, Wu T, Fang H, Zhou XJ, Ahn CW, Magder LS, Mohan C, Petri M (2012). Urinary vascular cell adhesion molecule, but not neutrophil gelatinase-associated lipocalin, is associated with lupus nephritis. J Rheumatol.

[45] Pitashny M, Schwartz N, Qing X, Hojaili B, Aranow C, Mackay M, Putterman C (2007). Urinary lipocalin-2 is associated with renal disease activity in human lupus nephritis. Arthritis Rheum.

[46] Yang CC, Hsieh SC, Li KJ, Wu CH, Lu MC, Tsai CY, Yu CL (2012). Urinary neutrophil gelatinase-associated lipocalin is a potential biomarker for renal damage in patients with systemic lupus erythematosus. J Biomed Biotechnol.

[47] Fournier T, Bouach N, Delafosse C, Crestani B, Aubier M (1999). Inducible expression and regulation of the alpha 1-acid glycoprotein gene by alveolar macrophages: prostaglandin E2 and cyclic AMP act as new positive stimuli. J Immunol.

[48] Sorensson J, Matejka GL, Ohlson M, Haraldsson B (1999). Human endothelial cells produce orosomucoid, an important component of the capillary barrier. Am J Physiol.

[49] Nakamura T, Board PG, Matsushita K, Tanaka H, Matsuyama T, Matsuda T (1993). Alpha 1-acid glycoprotein expression in human leukocytes: possible correlation between alpha 1-acid glycoprotein and inflammatory cytokines in rheumatoid arthritis. Inflammation.

[50] Ohara N, Hanyu O, Hirayama S, Nakagawa O, Aizawa Y, Ito S, Sone H (2014). Hypertension increases urinary excretion of immunoglobulin G, ceruloplasmin and transferrin in normoalbuminuric patients with type 2 diabetes mellitus. J Hypertens.

[51] Urade Y, Hayaishi O (2000). Biochemical, structural, genetic, physiological, and pathophysiological features of lipocalin-type prostaglandin D synthase. Biochim Biophys Acta.

[52] Hirawa N, Uehara Y, Ikeda T, Gomi T, Hamano K, Totsuka Y, Yamakado M, Takagi M, Eguchi N, Oda H, Seiki K, Nakajima H, Urade Y (2001). Urinary prostaglandin D synthase (beta-trace) excretion increases in the early stage of diabetes mellitus. Nephron.

[53] Otten MH, Cransberg K, van Rossum MA, Groothoff JW, Kist-van Holthe JE, Ten Cate R, Van Suijlekom-Smit LW (2010). Disease activity patterns in juvenile systemic lupus erythematosus and its relation to early aggressive treatment. Lupus.

