# Supplementary material for: International validation of a urinary biomarker panel for identification of active lupus nephritis in children
Source: Pediatr Nephrol. 2016 Sep 3;32(2):283–95. doi: 10.1007/s00467-016-3485-3 (PMC5203828; doi:10.1007/s00467-016-3485-3)
Supplement: Supplementary file 2 — Correlation between urine biomarkers in cohorts 1 and 2 (DOCX 88 kb) [file 467_2016_3485_MOESM2_ESM.docx]

**On-line resource 2 Correlation between urine biomarkers in cohorts 1 and 2**

| **Cohort 1** | **LPGDS** | **MCP-1** | **CP** | **AGP** | **VCAM-1** | **TF** |
| --- | --- | --- | --- | --- | --- | --- |
| **LPGDS** |  | 0.57 | 0.64 | 0.71 | 0.65 | 0.24 |
| **MCP-1** | 0.57 |  | 0.51 | 0.44 | 0.44 | 0.18 |
| **CP** | 0.64 | 0.51 |  | 0.67 | 0.44 | 0.39 |
| **AGP-1** | 0.71 | 0.44 | 0.67 |  | 0.63 | 0.41 |
| **VCAM1** | 0.65 | 0.44 | 0.44 | 0.63 |  | 0.30 |
| **TF** | 0.24 | 0.18 | 0.39 | 0.41 | 0.30 |  |
| **Cohort 2** | **LPGDS** | **MCP-1** | **CP** | **AGP** | **VCAM-1** | **TF** |
| **LPGDS** |  | 0.58 | 0.51 | 0.74 | 0.79 | 0.59 |
| **MCP-1** | 0.58 |  | 0.71 | 0.67 | 0.57 | 0.63 |
| **CP** | 0.51 | 0.71 |  | 0.87 | 0.63 | 0.91 |
| **AGP** | 0.74 | 0.67 | 0.87 |  | 0.82 | 0.92 |
| **VCAM-1** | 0.79 | 0.57 | 0.63 | 0.82 |  | 0.69 |
| **TF** | 0.59 | 0.63 | 0.91 | 0.92 | 0.69 |  |

Biomarker concentrations standardised to urinary creatinine. Spearman’s correlation tests used and correlation matrix including co-efficients (*r*) displayed.

Article title – International validation of a urinary biomarker panel for identification of active lupus nephritis in children

Journal name – Pediatric Nephrology

Author names – Smith EMD, Jorgensen AL, Midgley A, Oni L, Goilav B, Putterman C, Wahezi D, Rubinstein T, Ekdawy D, Corkhill R, Jones CA, Marks SD, Newland P, Pilkington C, Tullus K, Beresford MW.

Affiliation and e-mail address of the corresponding author - Dr. Eve Smith, University of Liverpool, e-mail: [esmith8@liverpool.ac.uk](mailto:esmith8@liverpool.ac.uk)
