# Supplementary material for: International validation of a urinary biomarker panel for identification of active lupus nephritis in children
Source: Pediatr Nephrol. 2016 Sep 3;32(2):283–95. doi: 10.1007/s00467-016-3485-3 (PMC5203828; doi:10.1007/s00467-016-3485-3)
Supplement: Supplementary file 3 — Urine biomarker concentrations (standardised to urinary creatinine) according to renal BILAG score in patients from the UK exploratory cohort. (DOCX 115 kb) [file 467_2016_3485_MOESM3_ESM.docx]

**On-line resource 3** **Urine biomarker concentrations standardised to urinary creatinine according to renal BILAG score in patients from the UK exploratory cohort.**

| **Biomarker** | **Renal BILAG score** | | | | ***p_c_-values* for comparisons^a^** | |
| --- | --- | --- | --- | --- | --- | --- |
|  | **A** | **B** | **D** | **E** | **A vs B** | **D vs E** |
| **LPGDS**  **(ngmgCr)** | 2476  [1958-  2994] | 1073  [565.1-  1897] | 312.1  [152.4-  617.3] | 215.3  [139.2-  583.8] | *0.79* | *1.00* |
| **MCP-1**  **(pgmgCr)** | 1657  [580.3-  2733] | 369.4  [175.4-  560.3] | 194.8  [111.1-  289.7] | 152.2  [96.05-  298.1] | *1.00* | *1.00* |
| **CP**  **(ngmgCr)** | 22970  [20521-  25419] | 3836  [1693-  20827] | 748.0  [505.9-  1150] | 627.3  [477.9-  968.7] | *1.00* | *1.00* |
| **AGP**  **(ngmgCr)** | 126005  [93109-  158901] | 18331  [680.1-  27789] | 311.9  [166.0-  774.2] | 250.0  [70.12-  594.7] | *0.133* | *1.00* |
| **VCAM-1^b^**  **(ngmgCr)** | 15.48  [8.18-  22.78] | 17.76  [8.82-  77.66] | 2.390  [1.17-  9.97] | 1.710  [0.49-  4.52] | *1.00* | *1.00* |
| **TF**  **(ngmgCr)** | 250673  [1457-  499890] | 7754  [694.3-  120772] | 1581  [422.0-  8067] | 659.0  [278.5-  1908] | *1.00* | *0.85* |
| **NGAL**  **(ngmgCr)** | 13.00  [6.30-  19.70] | 22.00  [12.20-  35.60] | 14.50  [7.10-  39.25] | 8.650  [5.60-  16.40] | *1.00* | *1.00* |

Biomarker concentrations standardised to urinary creatinine and expressed as median values and interquartile ranges. Mann Whitney U tests used to compare biomarker concentrations between patient groups. ^a^A Bonferroni adjustment was applied to account for multiple testing, corrected p-values are reported (p_c_), ^b^VCAM-1 measurement missing from 1 patient.

Article title – International validation of a urinary biomarker panel for identification of active lupus nephritis in children

Journal name – Pediatric Nephrology

Author names – Smith EMD, Jorgensen AL, Midgley A, Oni L, Goilav B, Putterman C, Wahezi D, Rubinstein T, Ekdawy D, Corkhill R, Jones CA, Marks SD, Newland P, Pilkington C, Tullus K, Beresford MW.

Affiliation and e-mail address of the corresponding author - Dr. Eve Smith, University of Liverpool, e-mail: [esmith8@liverpool.ac.uk](mailto:esmith8@liverpool.ac.uk)
